# Performance of Single and Concatenated Sets of Mitochondrial Genes at Inferring Metazoan Relationships Relative to Full Mitogenome Data

**DOI:** 10.1371/journal.pone.0084080

**Published:** 2014-01-08

**Authors:** Justin C. Havird, Scott R. Santos

**Affiliations:** 1 Department of Biological Sciences, Molette Biology Laboratory for Environmental and Climate Change Studies, Auburn University, Auburn, Alabama, United States of America; 2 Cellular and Molecular Biosciences Program, Auburn University, Auburn, Alabama, United States of America; University of Veterinary Medicine Hanover, Germany

## Abstract

Mitochondrial (mt) genes are some of the most popular and widely-utilized genetic loci in phylogenetic studies of metazoan taxa. However, their linked nature has raised questions on whether using the entire mitogenome for phylogenetics is overkill (at best) or pseudoreplication (at worst). Moreover, no studies have addressed the comparative phylogenetic utility of mitochondrial genes across individual lineages within the entire Metazoa. To comment on the phylogenetic utility of individual mt genes as well as concatenated subsets of genes, we analyzed mitogenomic data from 1865 metazoan taxa in 372 separate lineages spanning genera to subphyla. Specifically, phylogenies inferred from these datasets were statistically compared to ones generated from all 13 mt protein-coding (PC) genes (i.e., the “supergene” set) to determine which single genes performed “best” at, and the minimum number of genes required to, recover the “supergene” topology. Surprisingly, the popular marker COX1 performed poorest, while ND5, ND4, and ND2 were most likely to reproduce the “supergene” topology. Averaged across all lineages, the longest ∼2 mt PC genes were sufficient to recreate the “supergene” topology, although this average increased to ∼5 genes for datasets with 40 or more taxa. Furthermore, concatenation of the three “best” performing mt PC genes outperformed that of the three longest mt PC genes (i.e, ND5, COX1, and ND4). Taken together, while not all mt PC genes are equally interchangeable in phylogenetic studies of the metazoans, some subset can serve as a proxy for the 13 mt PC genes. However, the exact number and identity of these genes is specific to the lineage in question and cannot be applied indiscriminately across the Metazoa.

## Introduction

All metazoans (i.e., multicellular animals) possess a symbiotically derived genome within their mitochondria, the remnant of a bacterial endosymbiosis established early in eukaryotic evolution some two billion years ago [1], [2]. Known as the mitogenome, its size can vary considerably, from ∼2900 kilobases (kb) in muskmelons to 6 kb in *Plasmodium* [3]–[5], and some eukaryotes have lost it completely [6]. In metazoans, however, mitogenomes are relatively conserved in size (e.g., ∼14–20 kb) and generally encode the same 13 protein-coding (PC) and 24 structural RNA genes [7], [8]. Mitochondrial (mt) genes have arguably been some of the most popular and widely-utilized genetic loci in studies of animal phylogeography and phylogeny for the last three decades [9]–[11] due to the availability of “universal” polymerase chain reaction (PCR) primers (e.g., [12]), high copy number of mt genomes per cell, and assumed biological characteristics of near-neutrality, clonal inheritance, and a clock-like substitution rate [11]. Since elucidating the population structure and inferring the evolutionary histories of species is critical towards placing many aspects of their biology (e.g., physiology, ecology, conservation) into perspective, developing a deeper appreciation and understanding of the phylogenetic utility of mt genes has far-reaching implications.

As all mt genes generally reside on the same (typically circular) DNA molecule, it has often been implicitly or explicitly assumed that the mitogenome is a single “supergene”, with each gene possessing an identical, or highly similar, phylogenetic signal due to their linked nature [7]. Along these lines, some have suggested that sequencing and analyzing the entire mitogenome for particular phylogenetic questions is akin to pseudoreplication [13]. Furthermore, numerous studies have relied on single mt genes to infer evolutionary relationships (e.g., [14]) and the mt gene cytochrome oxidase subunit 1 (COX1) was recommended as the genetic marker for metazoan DNA barcoding [15], [16] and has been embraced by the Consortium for the Barcode of Life (http://www.barcodeoflife.org/). In this context and given that data from mt genes will continue to play a significant role in various biological disciplines, it is essential to: 1) identify whether each mt gene possesses the same phylogenetic signal and is thus interchangeable; 2) distinguish if particular genes perform better at representing the phylogenetic signal of the entire mitogenome; 3) determine just how many genes might be required to recreate the same topology as inferred from the complete mitogenome; and 4) establish whether any trends in #1–3 are lineage specific or can be generalized across metazoans. Answering these questions will shed light on mitogenome evolution, allow commentary on the robustness of previously published studies, and help shape future research directions.

Several studies have tested the assumption that a single PC gene can capture the phylogenetic signal of the entire mitogenome in clades where complete mitogenome sequences were available. In plethodontid salamanders, for example, ND4, ND2, and ND5 performed best at recreating the phylogeny inferred from complete mitogenomes [17]. However, ND4 was among the least phylogenetically informative genes when similar analyses were performed on caecilians [18], implying variation in the phylogenetic signal of the same mt gene exists among lineages. Likewise, phylogenetic performance of a particular mt gene may not extend to even closely related taxonomic groups: Duchêne et al. [19] found that while ND4 performed well at recreating a mitogenome phylogeny between whale species in Delphinidae, it did not for the delphinid genus *Orcinus*. The behavior of individual mt genes has also been assessed when recreating mitogenome phylogenies of well-known relationships (e.g., among vertebrate classes) with mixed results [20]–[23]. Lastly, as an example where multiple genes have been examined and/or concatenated, a phylogeny for the Insecta based on the entire mitogenome was recreated using five PC mt genes that performed best in individual gene analyses [24].

**Figure 1:**
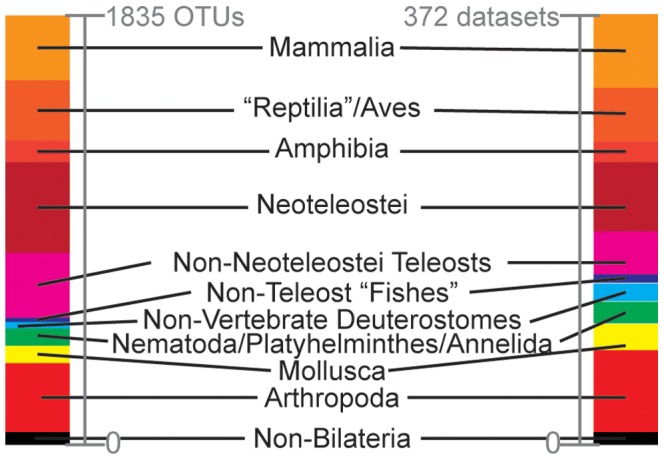
Data used in this study. Presented as proportions of all data, based on number of mitogenomes analyzed (left; 1835 mitogenomes in total) and number of lineages/datasets analyzed (right; 372 lineages in total).

While evaluating how individual mt PC genes (or some subset of them) perform in a phylogenetic context for specific groups and clades is interesting, no systematic survey has yet addressed this across a wide taxonomic range of metazoans, the largest eukaryotic group in which such genes are extensively utilized. Given the increased number of complete mitogenomes that have become available, such a study is now possible. Here, we computationally analyzed publically available mitogenome sequence data from across the major metazoan lineages (Fig. 1) to: 1) identify if particular mt PC genes perform “better” at inferring a topology comparable to that from all 13 PC genes (defined as the “supergene” set); 2) estimate the minimum number of mt PC genes required to statistically reproduce the same topology as the “supergene” set, and; 3) determine whether results varied within and/or between lineages or if identified trends could be generally applied. Along with this, parameters such as taxonomic level encompassed in the dataset, % divergence among taxa and number of operational taxonomic units (OTUs) within a clade, and whether analyses utilized amino acid or nucleotide data were investigated as potential drivers for variation in our results.

## Methods

### Data selection

Complete metazoan mitogenomes were downloaded from NCBI's organelle genome resource database between 11 June and 24 June 2010 (see below) and parsed into different hierarchical datasets (see Dataset S1). For example, all mitogenomes in the salamander genus *Ambystoma* were downloaded and utilized in three separate datasets representing different taxonomic “lineages”: *Ambystoma*, Salamandroidea, and Amphibia. Construction of each dataset was based on two criteria. Firstly, datasets were restricted to taxonomic ranks lower than phylum since mitochondrial DNA is fast-evolving in many metazoan lineages (but see [25]) and may not be appropriate for inferring deep (e.g., among all species in a phylum or between phyla) relationships (e.g., [26], [27]). Secondly, only lineages containing between 5 and 200 operational taxonomic units (OTUs) were selected since phylogenetic inference of < 5 OTUs is likely not representative of most phylogenetic datasets using mt genes, while > 200 OTUs were considered too computationally intensive. Notably, “OTU” is used instead of “species” since mitogenomes from subspecies (e.g., *Cervus nippon* subspecies) were included in some datasets. Generally, all OTUs with available and complete mitogenomes were used in phylogenetic analyses, with no OTUs spanning the different hierarchical datasets (i.e., outgroups were omitted from the individual phylogenetic analyses). However, several individual OTUs were excluded due to ambiguous or incomplete nucleotide sequences, errors in open reading frames following translation, or unconventional naming schemes that proved incompatible with our search strategies (see Dataset S1).

### Sequence alignments and phylogenetic analyses

The 13 protein-coding (PC) mt genes for each dataset were retrieved from NCBI's organelle genome resource database as both amino acid (AA) and nucleotide (nt) sequences and parsed by gene using MitoBank.pl v2.0 [28]. Amino acid sequences for each gene were aligned using ClustalW2 [29] under default settings. The nt sequences were subsequently aligned based on the AA alignments using the program tranalign in the EMBOSS suite [30]. To test if manual correction significantly improved alignments and/or impacted resulting phylogenies, all individual gene alignments for eight haphazardly selected datasets were inspected by eye, manually adjusted, and utilized in phylogenetic inference. The resulting phylogenies were not statistically different from ones inferred prior to manual correction based on Shimodaira-Hasegawa (SH) tests [31], suggesting such adjustments contributed little to no improvement to the alignments. Additionally, “supergene” phylogenies based on all 13 mt PC genes were visually compared to published phylogenies based on complete mitogenomes when possible (see Dataset S1 and Fig. S1).

Amino acid and nt alignments for individual mt genes were used to infer maximum likelihood (ML) phylogenies for a subset of the datasets (see Results) with the Pthreads version of RAxML v7.0.3 [32] at the Alabama Supercomputer Center (ASC) in Huntsville, Alabama. All datasets were analyzed using the PROTMIXWAGF model for AA data and the GTR + I + Γ model (GTRGAMMAI) for nt data, with 1000 rapid bootstrap replicates (‘-f a’ option) [33]. For the nt data, the third codon position was excluded from analyses due to potential mutational saturation and remaining data were partitioned by codon position, as is common in phylogenetic analyses of mitochondrial genes [34]–[39].

Phylogenies were also inferred for the datasets using concatenation of mt PC genes. The mt PC genes for each dataset were concatenated using the script fastacat.py (written by M. Robeson, Univ. of Colorado) and phylogenies generated under ML as above. All 13 protein-coding genes were concatenated to construct the “supergene” set. Additional concatenated alignments for all 372 datasets were also generated which included only the 12 longest PC genes, the 11 longest PC genes, and so on until the final alignment contained only the single longest PC gene. Genes lengths were ranked from longest to shortest (i.e., ND5, COX1, ND4, CYTB, ND2, ND1, COX3, COX2, ATP6, ND6, ND3, ND4L, and ATP8) based on a “typical” metazoan mitogenome (this length order was conserved, except for minor differences in basal metazoans, among 20 randomly chosen taxa across the datasets). Phylogenies were also inferred for a subset of the datasets using a concatenation of the three mt PC genes that individually performed “best” at recreating the “supergene” topology to determine whether they performed better than utilizing a concatenation of the three longest genes. Bayesian inference (BI) analyses were conducted with PhyloBayes v3.3 [40] at the ASC and utilized four chains per analysis to infer phylogenies from AA alignments for the larger (> 40 OTUs) datasets (see Results). The same model of evolution was used for BI analyses as in ML analyses and at least 2,000,000 generations were performed for each of the 13 AA alignments per dataset. The maximum discrepancy (max diff) between bipartitions was generally 0.3 or less (in 84% of analyses), supporting the probability that independent chains had reached a stable, matching point (see Dataset S1 for max diff values for each analysis). Average % divergence for each dataset was also calculated using the program infoalign in the EMBOSS suite [30]. All AA and nt alignments, tree files, and scripts used in this study are available as Dataset S2 and from The Santos Lab website (http://www.auburn.edu/~santosr/sequencedatasets.htm).

### Evaluation of phylogenies

For each dataset, phylogenies inferred from the 13 protein-coding genes (i.e., the “supergene” set) were statistically compared to those based on: 1) individual mt genes (for a subset of 63 datasets); 2) subsets comprised of the longest protein-coding genes (for all 372 datasets), and; 3) the three “best” performing mt genes (for a subset of 67 datasets). In some lineages (e.g., Bivalvia, Nematoda, and Platyhelminthes), nearly all taxa have lost the shortest protein-coding mt gene, ATP8. Given this, comparisons were made to the phylogeny inferred from the 12 protein-coding genes (see Dataset S1) for these lineages. To determine whether topologies derived from the mt gene subsets were statistically different from the “supergene” topology, comparisons were done via SH tests, as implemented in RAxML (‘-f h’ option). Changes in log-likelihood scores (ΔLn *L*) were also calculated between the “supergene” phylogeny and each of the phylogenies inferred from mt gene subsets. This was performed for phylogenies inferred using both ML and BI approaches as well as based on either AA or nt data. To complement the SH tests, the agreement metric [41] was also calculated using PAUP 4.0 [42] for a subset of the datasets (see Results). This metric computes the minimum number of leaves (i.e., OTUs) that need to be pruned from a phylogeny in order for it to be congruent with another phylogeny, and is therefore a reasonable accompaniment to the SH tests.

## Results

### Metazoan mitogenome datasets

Based on the selection criteria (see Methods), the protein-coding (PC) genes from 1865 mitogenomes constituting 372 metazoan lineages (i.e., monophyletic groups based on the organelle genome resources taxonomy tree of the National Center for Biotechnology Information (NCBI); Fig. 1, Dataset S1) were downloaded from NCBI's organelle genome resource database between 11 and 24 June 2010. The PC genes were parsed by gene identity, individually aligned, and used to infer ∼12,000 bootstrapped phylogenies under maximum likelihood (ML) (see Methods). The alignments and resulting phylogenies for all 372 datasets are available as Dataset S2 and from The Santos Lab website (http://www.auburn.edu/~santosr/sequencedatasets.htm) as a resource for addressing additional questions (e.g., agreement between mt and nuclear phylogenies). Furthermore, new mitogenomes are frequently deposited into NCBI (there has been a substantial increase of ∼1600 mitogenomes since our analyses) and the methodology presented here can serve as an outline for future work as novel datasets become available. As a “control” for our methodological approach, 46 datasets with published phylogenies based on complete mitogenomes (i.e., concatenated alignments of all 13 PC genes) from across the Metazoa were compared to phylogenies inferred here for the same datasets utilizing all 13 PC genes (i.e., the “supergene” set) (see Dataset S1). In general, our topologies were nearly identical to those in the originally published phylogenies, with exceptions typically involving situations where multiple outgroups were included in the published phylogenies (see Fig. S1).

### Performance of individual mt PC genes

**Figure 2:**
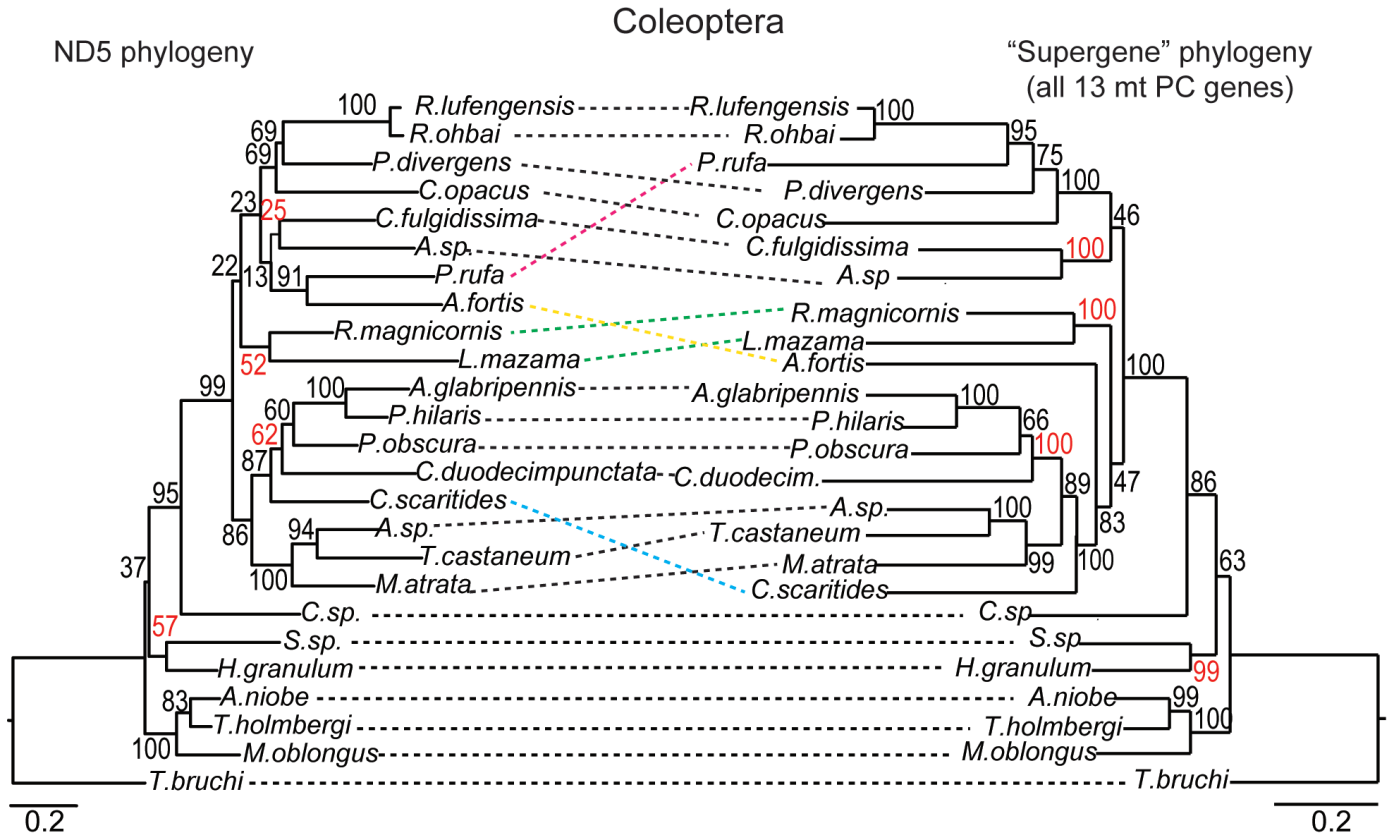
Example demonstrating discordance between the ND5 and the “supergene” phylogenies based on a specific lineage/dataset. Here, the two phylogenies for the Coleoptera were significantly different based on the Shimodaira-Hasegawa (SH) test and the ΔLn *L* between them is 264.6. For this example, a minimum of four mt protein-coding (PC) genes were required to infer a statistically indistinuishable topology to that of the “supergene” set (i.e., all 13 mt PC genes) when genes were selected by length, although the three mt PC genes that performed “best” (ND5, ND4, ND2) also inferred it. Clades/OTUs in the phylogeny showing different positions and/or relationships between the topologies are connected by colored lines, while those with the same positions and/or relationships are connected by black lines. Bootstrap support values (numbers at nodes) that increased noticeably for clades in the “supergene” topology as compared to ND5 are presented in red. Phylogenies analyzed here utilized amino acid data and were inferred via maximum likelihood (scale bars indicate replacements per site).

**Figure 3:**
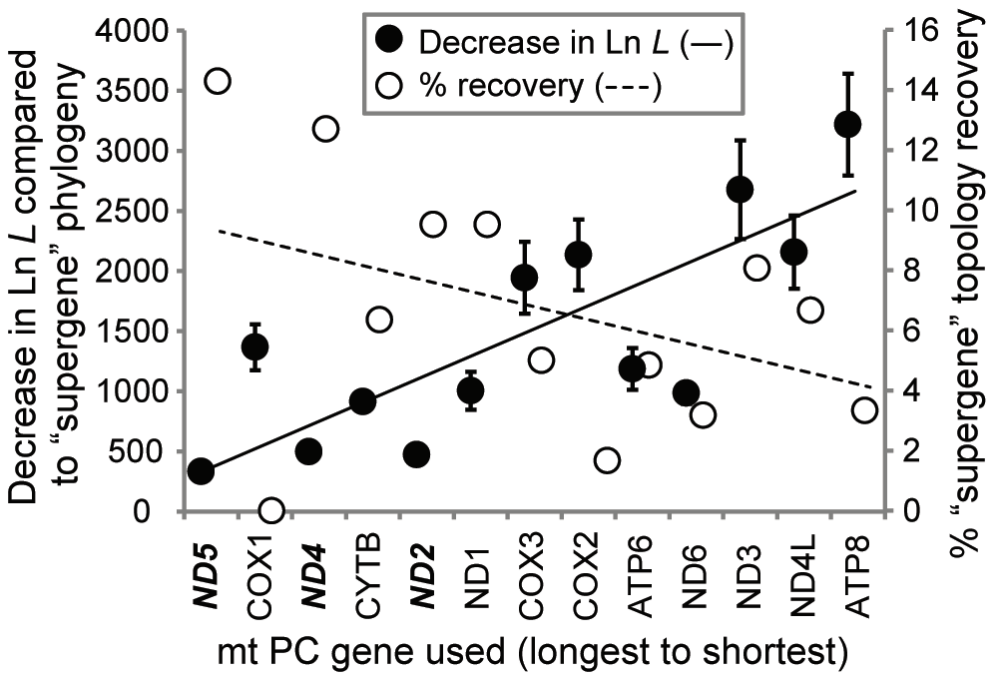
Comparisons between phylogenetic topologies inferred from single mt protein-coding (PC) genes relative to that of the “supergene” set. Presented as 1) decreases in Ln *L* (± S.E.M.) compared to the “supergene” phylogeny (i.e., all 13 PC genes, left *y*-axis) and 2) proportion of lineages where the “supergene” topology was recovered (right *y*-axis). The three “best” performing genes (i.e., ND5, ND2, ND1) are bolded and italicized along the *x*-axis. Single-gene phylogenies utilized amino acid data and were inferred via maximum likelihood for 63 datasets with > 40 OTUs and/or having previously published phylogenies (see text for details).

To determine whether and which particular mt PC genes were capable of reproducing the “supergene” (i.e., the complete set of 13 PC genes) topology, ML phylogenies were inferred from amino acid (AA) alignments of the individual mt PC genes and statistically compared to the “supergene” topology via Shimodaira-Hasegawa (SH) tests [31] for the 63 datasets with either > 40 OTUs or previously published mitogenome phylogenies (Dataset S1). Overall, phylogenies inferred from single mt PC genes generally had large decreases in log-likelihood (Ln *L*) relative to the “supergene” phylogeny and statistically different topologies for 54 of the 63 (86%) datasets (e.g., Fig. 2). While longer mt PC genes (i.e., ND2, ND4, ND5) individually tended to perform better than shorter ones (i.e., ND3, ND4L, ATP8), COX1, the second longest mt PC gene, surprisingly performed the poorest since it never reproduced the “supergene” topology in the datasets examined here and had a much greater decrease in Ln *L* when compared to the “supergene” phylogeny than expected based solely on its length (Fig. 3). In contrast, phylogenies inferred from ND5, ND4, or ND2 most frequently recovered a topology statistically indistinguishable from that of the “supergene”, with the smallest corresponding decreases in Ln *L* (Fig. 3).

### Performance of concatenated mt PC gene sets

**Figure 4:**
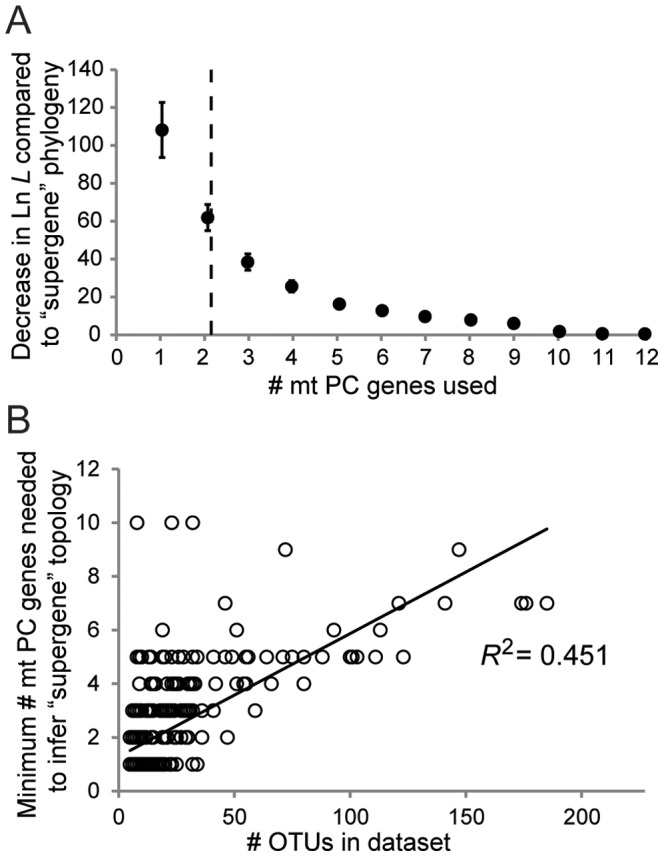
Comparisons between phylogenetic topologies inferred from concatenated mt protein-coding (PC) gene sets relative to that of the “supergene” set. A) Average decreases (± S.E.M.) in Ln *L* between phylogenies based on concatenated alignments of the longest mt protein-coding (PC) genes (from one to 12 genes) relative to the “supergene” (i.e., all 13 mt PC gene) phylogeny. The average gene number when this difference becomes significantly worse based on Shimodaira-Hasegawa (SH) tests is indicated by a vertical dashed line. B) Regression analysis of the minimum mt PC gene number required to infer a statistically indistinuishable topology to that of the “supergene” set relative to the number of OTUs per lineage. Phylogenies analyzed in A and B utilized amino acid data and were inferred for the 372 lineages examined in this study via maximum likelihood.

**Figure 5:**
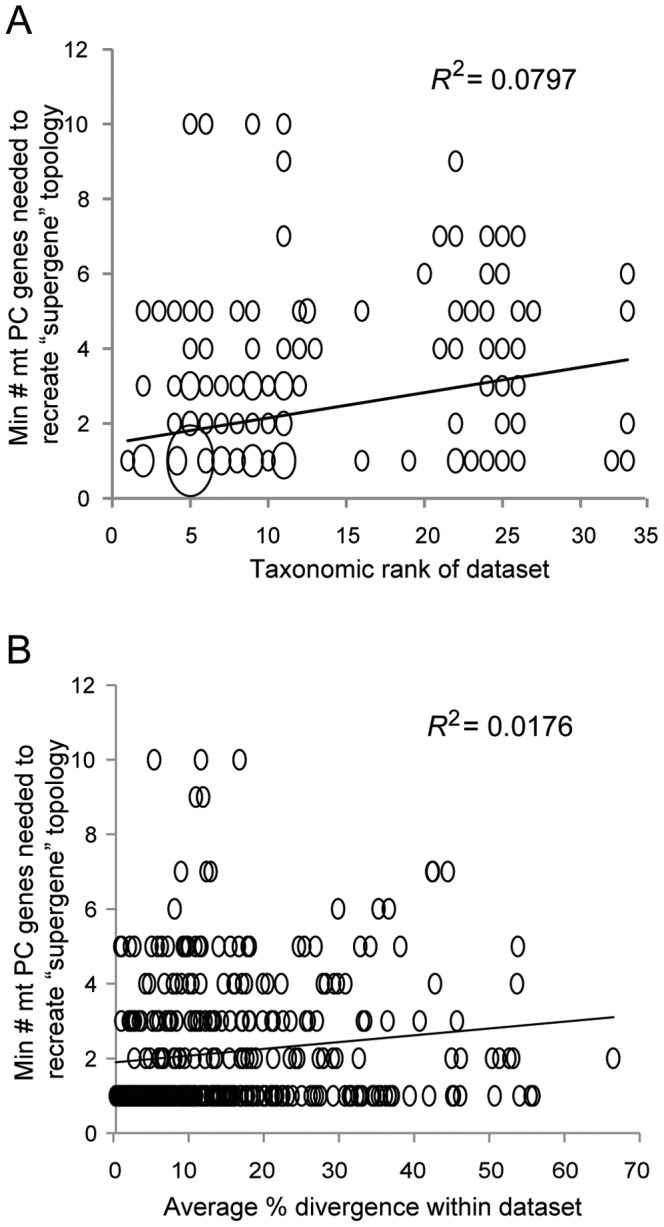
The minimum number of mt protein-coding (PC) genes required to infer the phylogenetic topology of the “supergene” set as a function of taxonomic rank and average % divergence. A) The number of mt protein-coding (PC) genes minimally required for inferring a statistically indistinuishable topology to that of the “supergene” set (i.e., all 13 mt PC genes) as a function of taxonomic rank of the lineage/dataset. A taxonomic rank of “1” along the *x*-axis corresponds to a within species phylogeny, “2” corresponds to within genus, etc. Subphylum was the highest rank analyzed, being assigned a taxonomic rank of 33. Taxonomic rank of each lineage followed the convention of NCBI as indicated in Dataset S1. The minimum number of mt protein-coding genes presented here are for phylogenies that utilized amino acid data and were inferred via maximum likelihood. Symbols of larger sizes represent of multiple datasets, with size being porportional to number of datasets. B) The number of mt protein-coding (PC) genes minimally required for inferring a statistically indistinuishable topology to that of the “supergene” set (i.e., all 13 mt PC genes) as a function of average % divergence of the lineage/dataset. The average % divergence was calculated using infoalign in EMBOSS. Minimum number of mt protein-coding genes presented here are for phylogenies that utilized amino acid data and inferred via maximum likelihood.

To estimate the minimum number of concatenated mt PC genes required to statistically reproduce the “supergene” topology, each of 12 individual phylogenies inferred from AA alignments of varying numbers of the longest mt PC genes (e.g., the single longest mt PC gene, the longest two mt PC genes, etc.) were statistically compared to the “supergene” topology using SH tests. Averaged across all 372 metazoan lineages, 2.16±0.09 (S.E.M.) of the longest mt PC genes statistically reproduced the “supergene” topology based on ML analyses of AA alignments and changes in Ln *L* scores (Fig. 4A). Similarly, 2.02±0.08 mt PC genes were sufficient to recover the “supergene” topology when nucleotide (nt) data were utilized (Fig. S2; Poisson regression, *z* = −1.34, *df* = 1, 371, *P* = 0.176 between AA and nt datasets). The “supergene” topology was recreated when using only the single longest gene (ND5) in 214/372 (58%) datasets, although such datasets had a significantly lower number of OTUs (averaging 8.6 vs. 34.2 OTUs, *P*<0.001, Student's unpaired *t*-test). The number of OTUs in a lineage was subsequently identified as a good predictor of the minimum number of genes required (Fig. 4B, *R* ^2^ = 0.451). However, taxonomic rank encompassed in the dataset and average % sequence divergence among taxa within a lineage were not good predictors of the minimum number of mt PC genes needed to infer the “supergene” topology (*R* ^2^ = 0.08 and 0.01, respectively, Fig. 5).

**Figure 6:**
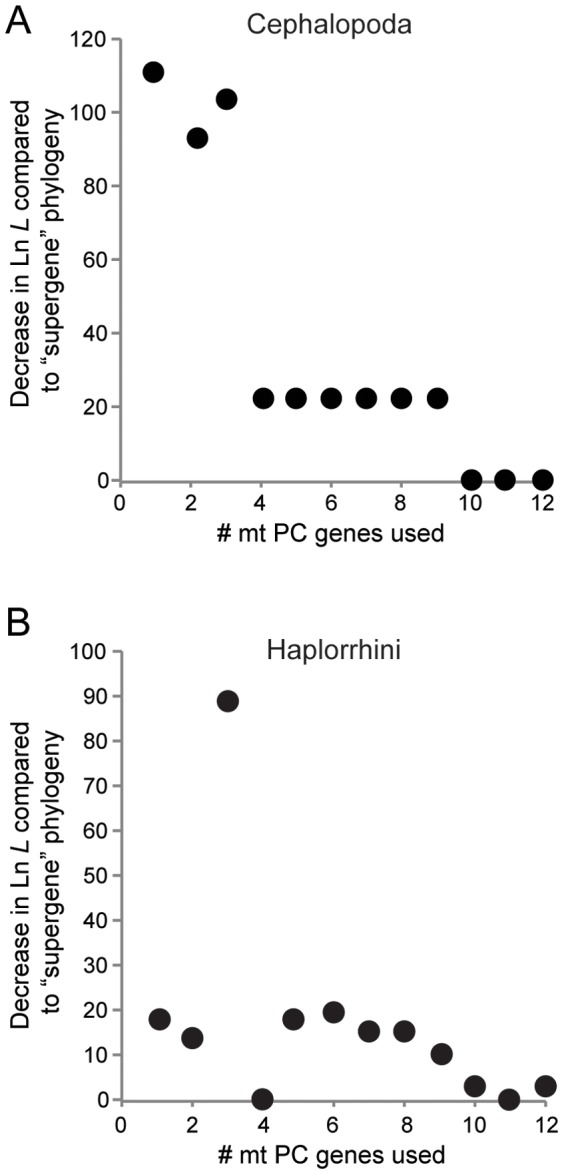
Examples demonstrating instances where the minimum number of mt protein-coding (PC) genes is either apparent or ambiguous. A) A phylogeny for the Cephalopoda where the number of mt protein-coding (PC) genes minimally required to infer a statistically indistinguishable topology to that of the “supergene” set (i.e., all 13 mt PC genes) is apparent. Shimodaira-Hasegawa (SH) tests indicated a minimum of four genes as being needed. The break is clearly indicated by the large decrease in Ln *L* compared to the “supergene” phylogeny when utilizing less than four genes or when utilizing four or more genes. This trend was typical for the majority of lineages examined (93.5% and 93.0% when utilizing amino acid and nucleotide data, respectively). B) A phylogeny for the Haplorrhini where the number of mt protein-coding (PC) genes minimally required to infer a statistically indistinguishable topology to that of the “supergene” topology is ambiguous. In this case, the SH tests indicated utilizing any combination other than three genes recovered the “supergene” topology, which is indicated by the large decrease in Ln *L* when just three genes are employed. In these cases, the minimum number of genes that statistically recreated the “supergene” topology was chosen (e.g., a single gene, NAD5, for Haplorrhini). This alternative trend was atypical of the data (6.5% and 7.0% of lineages when utilizing amino acid and nucleotide data, respectively). Comparisons presented in A and B are for phylogenies that utilized amino acid data and were inferred via maximum likelihood.

Notably, there was considerable variation in the estimated minimum number of mt PC genes needed to recover the “supergene” topology, sometimes corresponding to specific metazoan lineages. For instance, turtle lineages (specifically the Testudines, Testudinoidea, and Geoemydidae datasets) required the largest number of genes (10 total) to recreate the “supergene” phylogeny. Some lineages also required a larger number of genes despite having a relatively low number of OTUs. The turtle lineages are also an example of this since they possessed only 8–32 OTUs but required 10 genes (Fig. S3). Additionally, while the minimum number of genes required to infer the “supergene” topology could be clearly identified for 346 of 372 metazoan lineages examined here (∼93%), this was not the case for the other 26 (∼7%) lineages. In these situations, an intermediate, not minimum, number of mt PC genes produced a statistically different topology than the “supergene” topology (e.g., using 2 and 4 genes, but not 3, produced a statistically similar topology; Fig. 6A,B). For these cases, the minimum number of mt PC genes required to infer a topology that did not differ statistically from the “supergene” topology was utilized in the above estimates.

**Figure 7:**
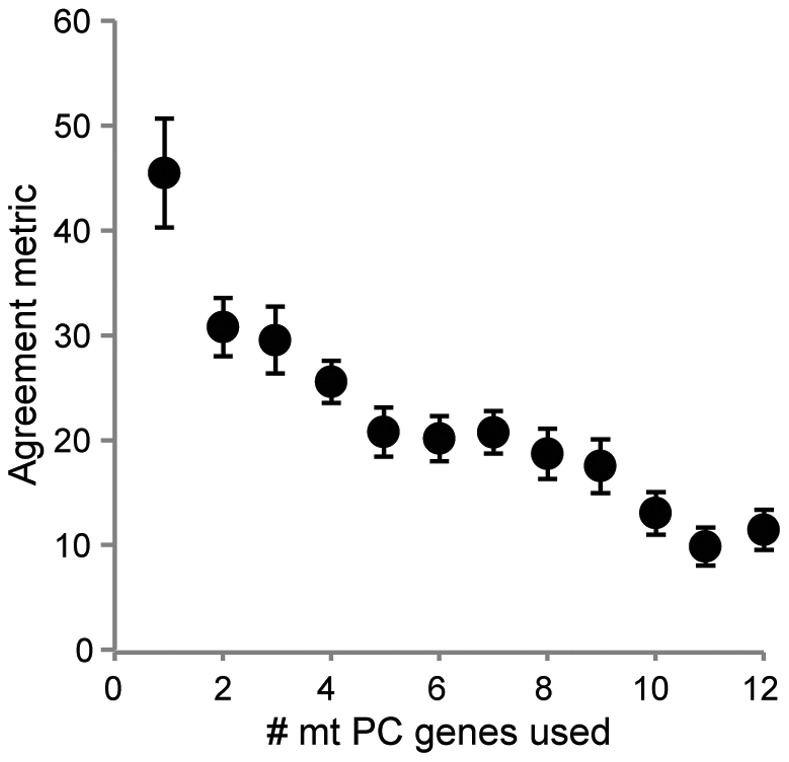
Agreement metrics using concatenated mt protein-coding (PC) gene sets. Agreement metrics (± S.E.M.) between topologies based on concatenated alignments of the longest mt protein-coding (PC) genes (from one to 12 genes) relative to the “supergene” (i.e., all 13 mt PC genes) topology. Phylogenies utilized amino acid data and were inferred via maximum likelihood for the 32 lineages with > 40 OTUs examined in this study.

**Figure 8:**
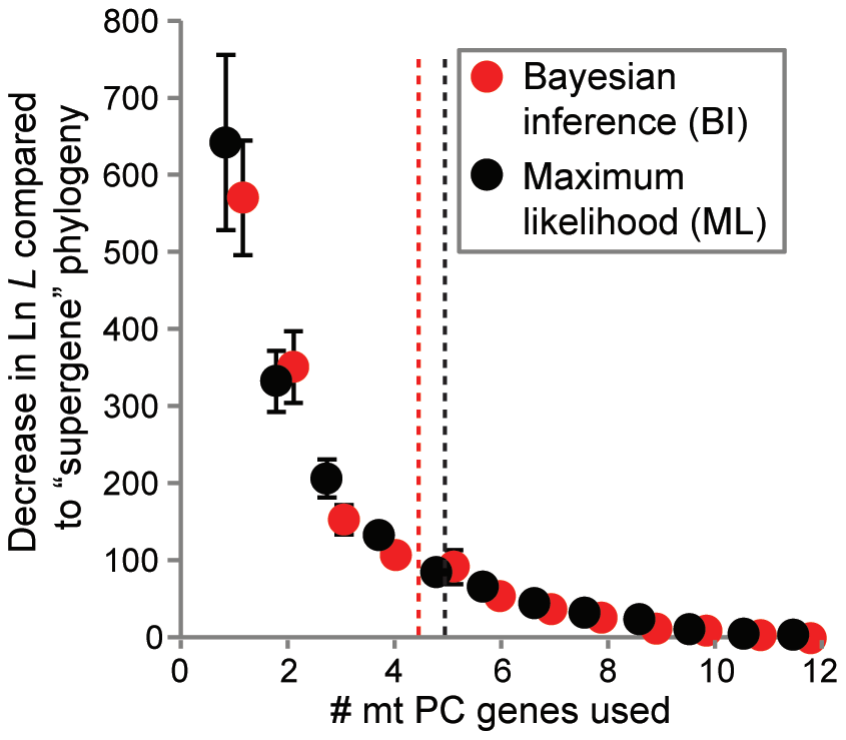
Comparisons between maximum likelihood (ML) and Bayesian inference (BI) on concatenated mt protein-coding (PC) gene sets relative to that of the “supergene” set. Average decreases (± S.E.M.) in Ln *L* between phylogenies based on concatenated alignments of the longest mt protein-coding (PC) genes (from one to 12 genes) relative to the “supergene” (i.e., all 13 mt PC genes) phylogeny. The average gene number when this difference becomes significantly worse based on Shimodaira-Hasegawa (SH) tests is indicated by a vertical dashed line (as in Figure 4A). Phylogenies utilized amino acid data and were inferred via either ML or BI for the 32 lineages with > 40 OTUs examined in this study. Overall, there was no difference in the minimum number of genes needed to recreate the “supergene” topology when using BI or ML methods (vertical dashed lines; Poisson regression, *z* = 0.74, *df* = 1,31, *P* = 0.458).

Since metazoan lineages with larger numbers of OTUs may be considered more representative of current and future phylogenetic datasets and because such lineages generally required more genes to recreate the “supergene” topology (see above), additional analyses were performed only on datasets with at least 40 OTUs (32 lineages total). These analyses included: 1) comparisons between ML phylogenies using the agreement metric [41] as an alternative to the SH test and 2) determining whether results inferred via Bayesian inference (BI) were consistent with those based on ML. In line with the trend identified above, more genes (5.16±0.23 and 4.81±0.25 when using AA and nt data, respectively: Poisson regression, z = −0.84, *df* = 1, 31, *P* = 0.40 between AA and nt data for the larger datasets) than the identified average across all datasets were needed for this subset of the data in order to infer the “supergene” topology based on ML and SH tests. Given that AA and nt data produced similar results, AA data were utilized in the below analyses. For the 32 lineages with > 40 OTUs, agreement metrics were consistent with results derived from SH tests and ΔLn *L* (Fig. 7). Phylogenies inferred under Bayesian inference (BI) also yielded a similar number of required genes as those derived from ML (Poisson regression, *z* = 0.74, *df* = 1,31, *P* = 0.458 between ML and BI approaches, Fig. 8), with SH-tests based on BI phylogenies yielding an average of 4.59±0.27 mt PC genes needed to recreate the “supergene” topology.

**Figure 9:**
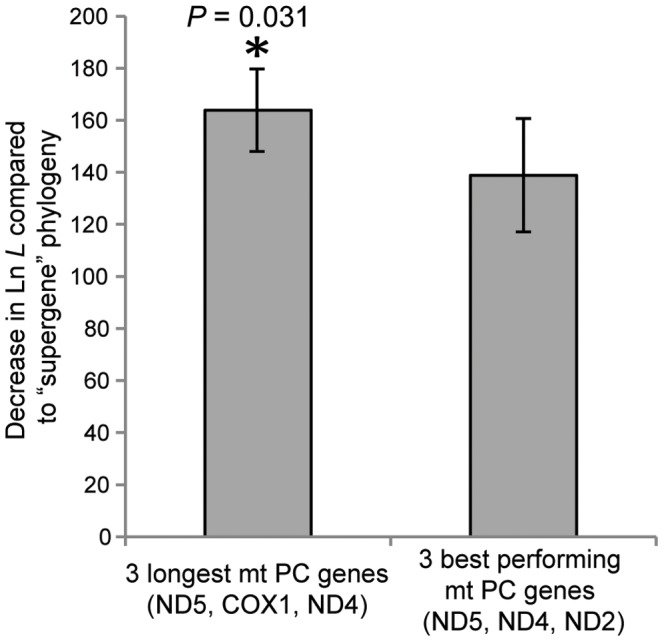
Comparison between selecting mt protein-coding (PC) genes by individual gene performance vs. by length. Utilizing the three mt protein-coding (PC) genes that individually performed “best” (based on Figure 3: ND5, ND4, ND2), rather than the three longest (i.e, ND5, COX1, and ND4) mt PC genes, resulted in a significantly lower decrease in Ln *L* (± S.E.M.) compared to the “supergene” (i.e., all 13 mt PC genes) topology (*t*-test, *t* = 2.21, *df* = 1, 66, *P* = 0.03). Additionally, the three “best” performing mt PC genes inferred a topology statistically indistinguishable from the “supergene” topology in 31% of datasets, whereas the three longest mt PC genes never inferred the “supergene” topology. Phylogenies utilized amino acid data and were inferred via maximum likelihood for the 67 lineages that originally required more than three mt PC genes when genes were chosen based solely on length (these included the 32 lineages with > 40 OTUs).

To determine whether subsets of mt PC genes chosen based on individual gene performance (see above) could recreate the “supergene” topology with higher success than when genes were chosen based solely on length, phylogenies were inferred from a concatenation of the three “best” performing mt PC genes (i.e., ND5, ND4, and ND2, see above). This was done for the 67 datasets that originally required more than three mt PC genes when those genes were selected by length (these included the 32 datasets with > 40 OTUs). In these cases, phylogenies inferred from the three “best” genes performed significantly better than ones from the concatenation of the three longest mt genes (i.e., ND5, COX1, and ND4), as measured via decreases in Ln *L* when compared to the “supergene” phylogenies (an average improvement of 25 Ln *L* units, *t*-test, *t* = 2.21, *df* = 1, 66, *P* = 0.03 between the “best” vs. longest three genes, Fig. 9). Phylogenies based on these three “best” genes also inferred the “supergene” topology in 31% of these datasets (as determined by SH tests) while those based on the three longest genes never produced a statistically indistinguishable topology to that of the “supergene” set.

## Discussion

### Individual gene performance

Although single-mt gene phylogenies generally performed poorly in the analyses presented here, some mt protein-coding (PC) genes were significantly better at reproducing a topology consistent with that of the “supergene” set, with ND2, ND4, and ND5 being the most likely to do so (Fig. 3). These results support previous studies [21], [22] identifying these genes as “very good” and “medium” for recreating well-established vertebrate relationships based on a low number of exemplar sequences. Surprisingly, we found COX1 performed among the worse at recreating the “supergene” topology, in contrast to previous studies [19], [21], [22], [24]. For example, COX1 performed well for closely related whales in Delphinidae and *Orcinus* [19] and while phylogenies inferred solely on COX1 for these specific lineages were not examined here, no single mt gene was capable of recreating the “supergene” topology for whales/even-toed ungulates (Cetartiodactyla) or baleen whales (Mysticeti), implying single mt genes may not be adequate for inferring phylogenetic relationships among these groups in general.

Why did COX1 perform poorly here in contrast to previous studies? Two significant differences between these previous studies and the analyses conducted here are the taxa in question and the taxonomic “breadth” under examination. In our study, all major metazoan lineages were analyzed, whereas in previous studies, single lineages or exemplar relationships were explored. Thus, while COX1 may provide applicable data for particular lineages [19], [24] or for recovering fairly deep relationships among a limited number of taxa (e.g., relationships among vertebrate classes [21], [22]), it apparently performs poorly overall for metazoan lineages in general. Taken together, COX1 possesses phylogenetic signal that may not be representative of the other mt genes in general and researchers should be cautious when basing hypotheses regarding the evolution of metazoan lineages solely on phylogenies derived from sequence data of this specific gene.

Although gene length was generally a good predictor of performance, among the longest genes those encoding proteins of the NADH dehydrogenase subunits (ND) tended to outperform those encoding the cytochrome carriers (COX, CYTB). One hypothesis, consistent with the results presented here, is that functionally different classes of mt genes possess their own distinctive phylogenetic signals due to varying evolutionary and/or selection pressures. Such a situation is apparent when comparing previous studies where comparable phylogenies were generated from different functional classes of genes. For example, within the Mollusca, phylogenies based on mt protein-coding genes recover a monophyletic Opisthobranchia [43]–[45], while those based on nuclear and mt ribosomal genes inferred the same group as being paraphyletic [46]–[48]. Here, given that ND genes are numerically dominant in the metazoan mitogenome (i.e., 7 of 13 protein-coding genes), it is not hard to imagine their phylogenetic signal exerting the greatest influence on the overall “supergene” topology, thus offering an explanation to why they tend to outperform other genes such as COX1. Another explanation is that COX/CYTB are more conserved than NAD genes [49], and therefore NAD genes may provide additional resolution that would be representative of the phylogenetic signal found in the “supergene” set. More importantly, the results of these single-gene analyses imply that despite their linked nature, not all mt genes are interchangeable in phylogenetic analyses.

### Performance of concatenated gene sets

The data presented here suggest a select subset of mt genes can serve as a proxy for all 13 PC mt genes when inferring complex metazoan phylogenies that include many OTUs. Moreover, these were not necessarily the longest mt genes. This demonstrates that sequencing more loci, one typical answer when addressing phylogenetic questions, may not produce a significantly better topology. In support of this, increasing the number of taxa has been found to be more effective than the inclusion of additional sequence data for taxa already present in data matrices [50]–[54]. Furthermore, in cases where an intermediate, not minimum, gene number produced a statistically different topology than the “supergene” set (7% of the datasets), inclusion of additional genes resulted in a conflicting topology. For example, while the “supergene” topology for camels (the Camelidae lineage) was recapitulated when analyzing only ND5, adding COX1 to the data matrix led to a significantly different (and conflicting) result. Such “rouge genes” could be the results of alternative strand-biases [55] or other yet unknown factors. However, these cases clearly demonstrate that the inclusion of more data is not only unnecessary, but also has the potential of producing significantly “worse” phylogenies in the process.

When all 372 datasets are considered, only the ∼2 longest mt PC genes, on average, were needed to infer a topology statistically indistinguishable to the “supergene” topology based on all 13 mt PC genes. However, it should be noted that the high number (214/372; 58%) of datasets 1) requiring only ND5 to recreate the “supergene” topology and 2) with a low number of OTUs, biases this estimate. Support for this comes from the fact that for datasets with > 40 OTUs, the estimate increased to an average of ∼5 mt PC genes (∼60% of the sequence data from all 13 mt PC genes). Likewise, greater numbers of mt PC genes were required for some specific lineages (e.g., turtles) than were estimated based solely on number of OTUs. In any case, given that single mt PC genes are usually not representative of the mitogenome while a fraction of them provides an adequate proxy supports the argument that, in most instances, entire mitogenome sequences are unnecessary towards resolving phylogenetic relationships [7], [19], [56].

Taxonomic rank was not a good predictor of the estimated minimum number of genes required to reproduce a topology consistent with that of the “supergene” set. This supports the idea that the traditional Linnaean classification hierarchy is arbitrary and may not reflect actual evolutionary relationships [57]. Along with this, average sequence divergence among taxa in a dataset was also a poor predictor for the estimated minimum number of genes. Finally, the identification of outlier lineages, such as the turtles, may represent interesting cases warranting closer examination. In this context, the metazoan mitogenome datasets assembled for this study will allow others to further explore the biological, evolutionary and/or methodological drivers (e.g., variable mutation rates, time since origin, incomplete taxon sampling, etc.) behind these particular instances. For example, reverse strand-bias in the nucleotide composition of mt PC genes has evolved multiple times in arthropods and been shown to significantly impact phylogenetic reconstruction [55]. Determining and utilizing gene(s) that minimize such biases would also influence the inference of the “supergene” topology. In this context, the methods employed here can be easily adapted to investigate the above situations, lineages not currently represented in the datasets due to a lack of data, or alternative combinations and concatenations of mt genes (e.g., chosen at random or based on their frequency of use from the literature).

## Conclusions

Here, we examined the performance of individual mt PC genes as well as concatenated loci across all major metazoan lineages in recreating the topology inferred from the entire mt protein-coding gene set. From a phylogenetic perspective, not all mt PC genes were found to be equally representative of the complete set and a single gene cannot serve as an adequate substitute for many phylogenetic inferences. Moreover, COX1, probably the most commonly utilized mt PC gene in studies of metazoans [58], [59], tends to perform poorly in a phylogenetic context. Importantly, sequencing the entire mitogenome was not necessary for any of the lineages examined; instead, the number and specific genes required to infer the “supergene” topology are lineage-specific. Although several mt loci are needed to serve as a proxy for all mt PC genes, there is little dispute that adding unlinked markers in the form of nuclear loci may produce a more robust and accurate topology since phylogenetic relationships can be masked by the evolutionary history of single genes, or in this case, multiple closely linked genes [60]. As genomic-scale phylogenies (i.e., phylogenomics) thought to represent the “true” evolutionary history between species become more common, similar questions regarding the phylogenetic signal in individual loci or groups of loci of interest and potential conflict among them [61]–[63] will need to be addressed.

## Supporting Information

Figure S1 **Comparisons between the “supergene” topology inferred here and 46 previously published mitogenome phylogenies (see Dataset S1).** The similarity of the topologies was assesed qualitatively and then converted to a score of “1” (not similar) to “5” (identical; see Dataset S1 for notes on comparisons). To achieve a score of “5” (which was given in 16 out of 46 comparisons), all taxa shared between both studies must have had identical relationships (i.e., the same topology). To achieve a score of “4” (which was given in 23 out of 46 comparisons), all shared taxa had to have identical relationships except for one to three clades at the tips of the trees involving no more than five taxa. A score of “3” (which was given in 6 out of 46 comparisons) were assigned to comparisons where shared taxa had the same relationships among major lineages, but greater than three clades at the tips of the tree showed differences, involving no more than eight taxa. A single comparison received a score of “2” because while all major lineages were recovered, the relationships between them were not. Scores of “1” were reserved for comparisons where a minority of the major lineages were recovered in our analyses. Similarity between the topologies was related to the proportion of taxa shared between the two phylogenies. For example, published phylogenies often had multiple outgroups that were not present in our analyses. Furthermore, our analyses often contained dozens of taxa for particular clades that may have been represented by a single or few exemplar taxa in the published studies. These account for all comparisons receiving scores less than a “4”. “Supergene” phylogenies utilized amino acid data and were inferred via maximum likelihood for the 46 lineages examined in this comparison.
(TIF)

Figure S2 **Comparisons between using amino acid (AA) or nucleotide (nt) data from mt protein-coding (PC) genes.** Average decreases (± S.E.M.) in Ln *L* between phylogenies based on concatenated alignments of the longest mt protein-coding (PC) genes relative to the “supergene” (i.e., all 13 mt PC gene) topology, as in Figure 4A. Phylogenies utilized either amino acid or nucleotide data and were inferred via maximum likelihood for all 372 datasets. Overall, there was no difference in the minimum number of genes required to infer a statistically indistinuishable topology to that of the “supergene” set between using amino acid or nucleotide data (vertical dashed lines; Poisson regression, *z* = −1.34, *df* = 1, 371, *P* = 0.176 between amino acid and nucleotide datasets).
(TIF)

Figure S3 **Residuals from** **Fig. 4B** **.** Outlier datasets requiring more mt protein-coding genes than the average predicted by the number of OTUs in the dataset have residuals < 0 while those requiring less than the predicted have residuals > 0. The turtle lineages are highlighted as an outlier (see text for details).
(TIF)

Dataset S1 **Summary of data utilized in this study.** First spreadsheet contains information on all 372 datasets used in the current study, including details of phylogenies built for each analyses. Second spreadsheet contains a summary of problematic OTUs (see Methods).
(XLS)

Dataset S2 **Data files utilized and generated in this study.** All AA and nt alignments, tree files, and scripts used in this study. Found at http://www.auburn.edu/~santosr/sequencedatasets.htm.
(DOCX)
