# Supplementary figures and images for: Performance of Single and Concatenated Sets of Mitochondrial Genes at Inferring Metazoan Relationships Relative to Full Mitogenome Data
Source: PLoS One. 2014 Jan 8;9(1):e84080. doi: 10.1371/journal.pone.0084080 (PMC3891902; doi:10.1371/journal.pone.0084080)

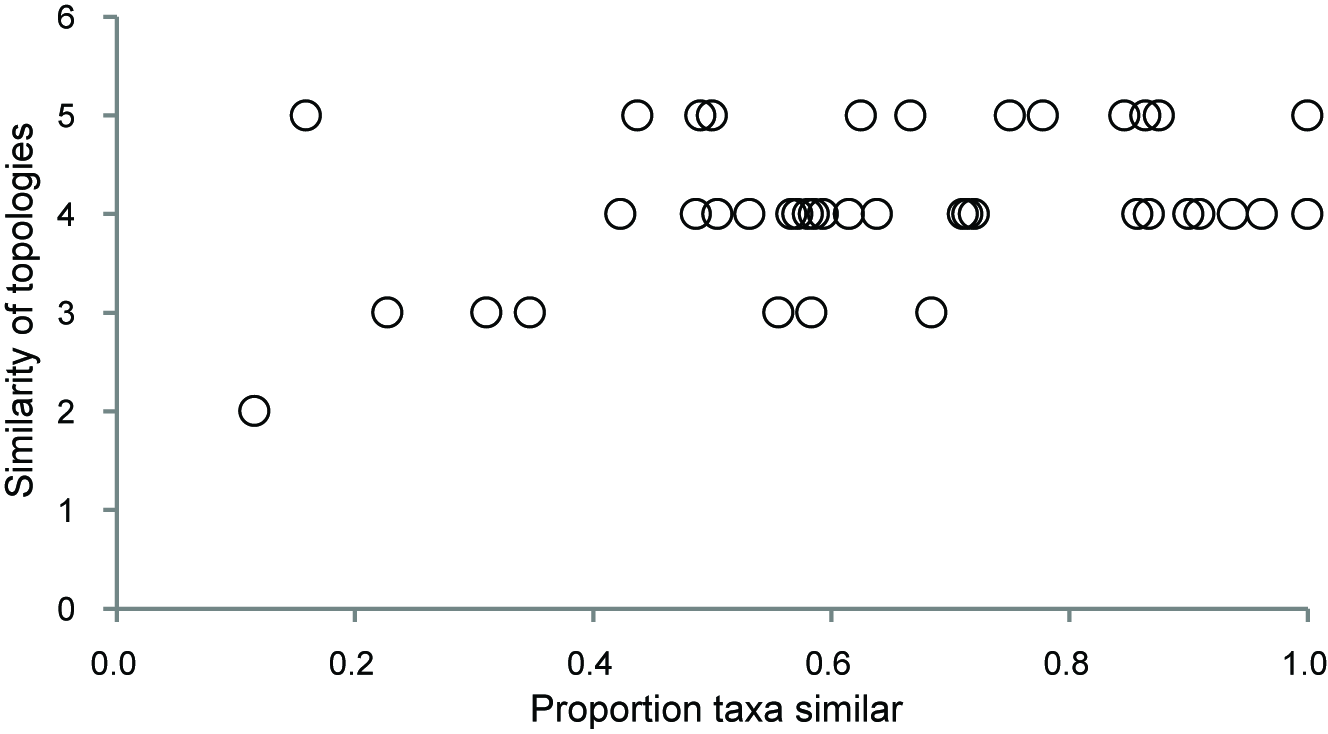

Supplement: Figure S1 — Comparisons between the “supergene” topology inferred here and 46 previously published mitogenome phylogenies (see Dataset S1). The similarity of the topologies was assesed qualitatively and then converted to a score of “1” (not similar) to “5” (identical; see Dataset S1 for notes on comparisons). To achieve a score of “5” (which was given in 16 out of 46 comparisons), all taxa shared between both studies must have had identical relationships (i.e., the same topology). To achieve a score of “4” (which was given in 23 out of 46 comparisons), all shared taxa had to have identical relationships except for one to three clades at the tips of the trees involving no more than five taxa. A score of “3” (which was given in 6 out of 46 comparisons) were assigned to comparisons where shared taxa had the same relationships among major lineages, but greater than three clades at the tips of the tree showed differences, involving no more than eight taxa. A single comparison received a score of “2” because while all major lineages were recovered, the relationships between them were not. Scores of “1” were reserved for comparisons where a minority of the major lineages were recovered in our analyses. Similarity between the topologies was related to the proportion of taxa shared between the two phylogenies. For example, published phylogenies often had multiple outgroups that were not present in our analyses. Furthermore, our analyses often contained dozens of taxa for particular clades that may have been represented by a single or few exemplar taxa in the published studies. These account for all comparisons receiving scores less than a “4”. “Supergene” phylogenies utilized amino acid data and were inferred via maximum likelihood for the 46 lineages examined in this comparison. (TIF) [file pone.0084080.s001.tif]

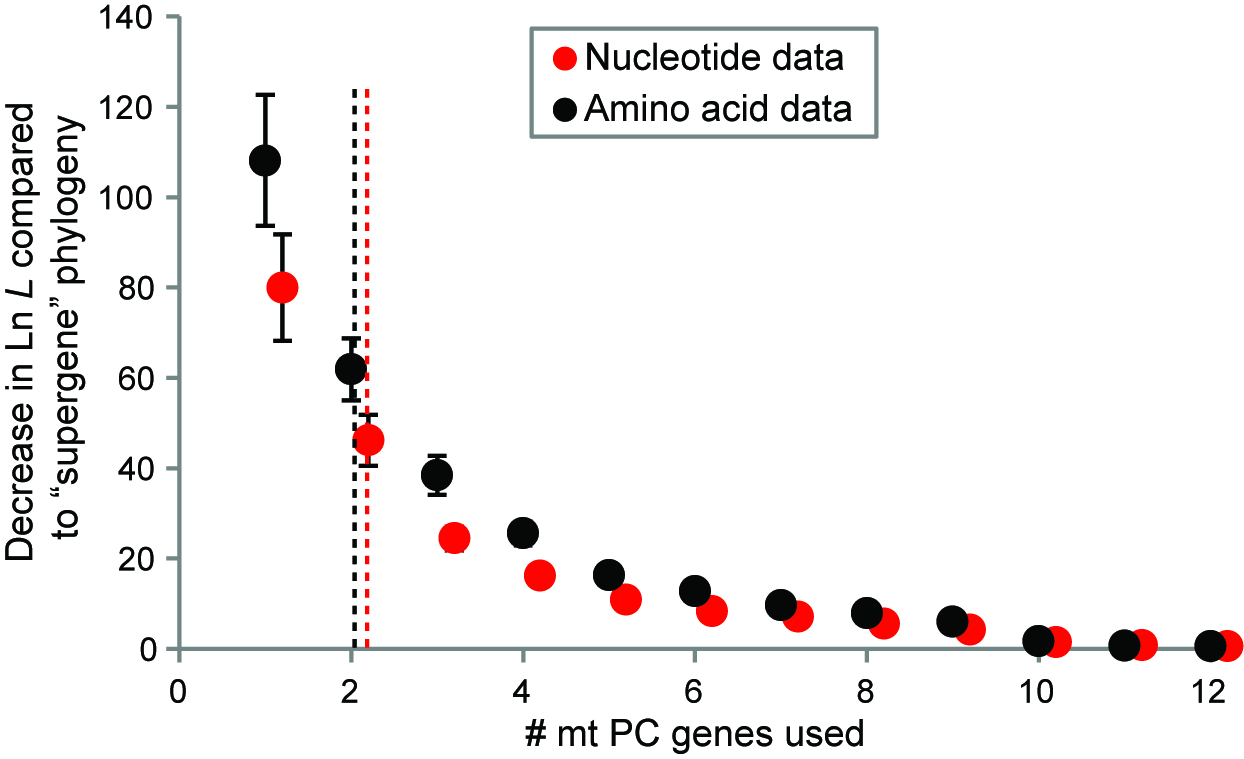

Supplement: Figure S2 — Comparisons between using amino acid (AA) or nucleotide (nt) data from mt protein-coding (PC) genes. Average decreases (± S.E.M.) in Ln L between phylogenies based on concatenated alignments of the longest mt protein-coding (PC) genes relative to the “supergene” (i.e., all 13 mt PC gene) topology, as in Figure 4A. Phylogenies utilized either amino acid or nucleotide data and were inferred via maximum likelihood for all 372 datasets. Overall, there was no difference in the minimum number of genes required to infer a statistically indistinuishable topology to that of the “supergene” set between using amino acid or nucleotide data (vertical dashed lines; Poisson regression, z = −1.34, df = 1, 371, P = 0.176 between amino acid and nucleotide datasets). (TIF) [file pone.0084080.s002.tif]

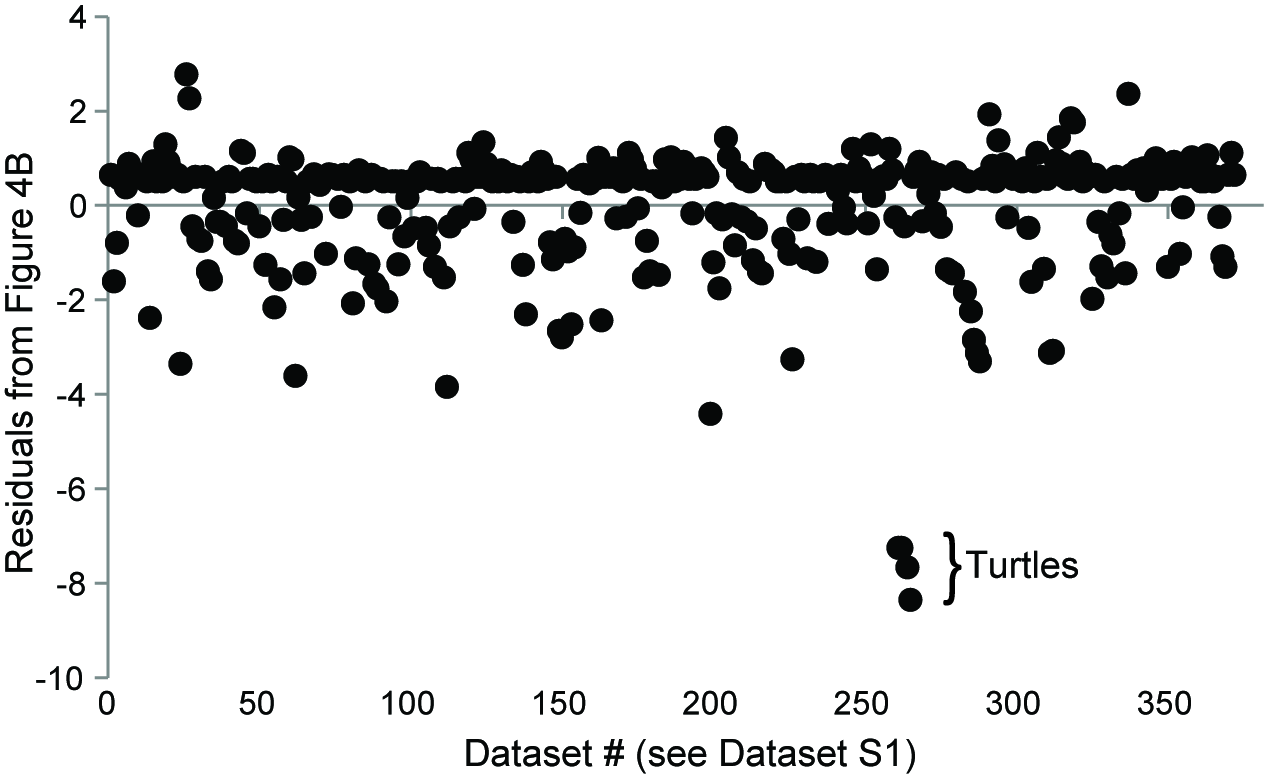

Supplement: Figure S3 — Residuals from Fig. 4B . Outlier datasets requiring more mt protein-coding genes than the average predicted by the number of OTUs in the dataset have residuals < 0 while those requiring less than the predicted have residuals > 0. The turtle lineages are highlighted as an outlier (see text for details). (TIF) [file pone.0084080.s003.tif]
