# Supplementary material for: Performance of Single and Concatenated Sets of Mitochondrial Genes at Inferring Metazoan Relationships Relative to Full Mitogenome Data
Source: PLoS One. 2014 Jan 8;9(1):e84080. doi: 10.1371/journal.pone.0084080 (PMC3891902; doi:10.1371/journal.pone.0084080)
Supplement: Dataset S2 — Data files utilized and generated in this study. All AA and nt alignments, tree files, and scripts used in this study. Found at http://www.auburn.edu/~santosr/sequencedatasets.htm. (DOCX) [file pone.0084080.s005.docx]

**Dataset S2.**

**Data files utilized and generated in this study.** All AA and nt alignments, tree files, and scripts used in this study. Found at <http://www.auburn.edu/~santosr/sequencedatasets.htm>
